# The effects of exercise with nicotine replacement therapy for smoking cessation in adults: A systematic review

**DOI:** 10.3389/fpsyt.2022.1053937

**Published:** 2022-11-24

**Authors:** Hui Chen, Yang Yang, Hanna Miyai, Chenju Yi, Brian G. Oliver

**Affiliations:** ^1^Research Centre, The Seventh Affiliated Hospital of Sun Yat-sen University, Shenzhen, China; ^2^Faculty of Science, School of Life Sciences, University of Technology Sydney, Ultimo, NSW, Australia; ^3^Respiratory Cellular and Molecular Biology, Woolcock Institute of Medical Research, The University of Sydney, Glebe, NSW, Australia

**Keywords:** cigarette smoking, quitting smoking, smoking abstinence, behavioural therapy, clinical trial

## Abstract

**Objective:**

This systematic review aimed to evaluate the efficacy of exercise programmes with nicotine replacement therapy (NRT) for smoking cessation in adults.

**Introduction:**

Nicotine addiction is mediated by dopamine. Exercise can also activate the dopamine reward system. Therefore, exercise may effectively facilitate NRT to reduce cigarette cravings and withdrawal symptoms.

**Inclusion criteria:**

Clinical trials between 2000 and 2022 used exercise protocols of any intensity for smoking cessation, in current smokers or recent quitters of both genders, aged 18–70, without severe diseases and pregnancy. Mental disorders were not excluded, as exercise can improve mental health status. Therefore, it may be as effective among people with mental health issues as the general population in preventing nicotine cravings and supporting abstinence.

**Methods:**

Four databases (PubMed, Embase, Cochrane, and Medline) were searched for papers in English using the terms “nicotine replacement therapy’, “exercise,” and “smoking cessation.” Titles and abstracts were screened for potentially eligibility before full texts were reviewed. Sample size, gender, study duration, and age was then extracted. The certainty of the evidence was assessed using Joanna Briggs Institute’s (JBI’s) GRADE approach.

**Results:**

Seventeen studies were identified with a total of 3,191 participants. Three studies are not a randomised control study. There was moderate-high quality evidence that exercise can aid NRT in promoting smoking cessation in the short term. Several studies reported temporary reductions in cravings; however, only one trial reported a decrease in cigarette consumption due to exercise intervention and one demonstrated increased smoking abstinence at 1 year of the intervention.

**Conclusion:**

Exercise with NRT aids smoking cessation in the short term, but no evidence suggests its efficacy in the long term when combined. Future trials should include larger sample sizes and strategies to increase exercise adherence.

## Introduction

Tobacco smoking is the leading cause of preventable death and illness across the globe, causing more than 8 million deaths each year (1). Over 50% of these deaths occur in people between the ages of 30 and 69 (2). Based on current smoking trends, it has been estimated that 450 million adults will die from smoking between the years 2000–2050. The ‘National Drug Strategy Household Survey 2019’ (3) by the Australian Institute of Health and Welfare reports that 70% of current adult smokers would like to quit (4). However, smoking cessation is difficult, as it can take more than 30 attempts before a smoker is successful (5). Not supervising, approximately 66% of Australian smokers are classified as unsuccessful quitters (6). In 2019, the prevalence rate of smoking was 14.5% among the general adult population, which was 26.8% among adults with mental illness (7). The higher smoking rate among individuals with mental illness is unclear (8). However, having a broad range of effective cigarette cessation options may encourage more quitting attempts among smokers, especially being more beneficial for reducing the health burden of smoking-related illnesses and disabilities (8).

When undergoing quit attempts, smokers are likely to experience cigarette cravings due to nicotine addiction. This is typically managed using pharmacotherapies, such as nicotine replacement therapy (NRT). NRT is, in fact, a gold standard intervention for smoking cessation, which can increase the rate of quitting by 50–70%, regardless of the setting (9). NRTs have been designed to mimic the nicotine delivery from inhaling cigarette smoke. Higher doses of NRTs make a rewarding feeling and suppress the craving due to nicotine abstinence from cigarette smoke (10). However, its effectiveness is limited.

There is a negative correlation between physical activity and cigarette consumption, which may ultimately lead to smoking cessation (11). It is possible that exercise can provide further relief from cravings and withdrawal symptoms in ways that NRT is unable to do (12). Increased brain “happy” neurotransmitters, such as beta-endorphins, serotonin, and dopamine, are partly responsible for “exercise euphoria” (13). Thus, exercise, particularly resistance training, is able to manage psychological risk factors leading to relapses, such as stress, anxiety, depression, fatigue, poor concentration, irritability and restlessness (12, 14–16). Indeed, short bouts of exercise have been shown to alleviate withdrawal symptoms (15, 17). There have been several proposed mechanisms, such as exercise activating the same reward pathway as nicotine, which also drives the craving for other addictive substances (15). It has also been suggested that exercise can alleviate cravings by reducing levels of stress hormones, such as cortisol and adrenaline (15, 18). These mechanisms differ significantly from NRT, highlighting the potential of a greater success rate when combining exercise and NRT for smoking cessation (19).

Notwithstanding the potential of exercise to aid smoking cessation and reduce relapse, exercise has several other fitness benefits when maintained regularly for the general population, as well as for smokers (20). Exercise has been shown to attenuate smoking cessation-induced weight gain, which has been a significant factor preventing many smokers from quitting smoking, especially younger age ones (21). Resistance training, such as weight training, improves lung function, blood lipids and glucose profiles, health risk factors associated with smoking and thus risk of several chronic diseases (16). Both aerobic- and resistance-based interventions can reduce the risk of several prevalent cancer types initiated by smoking, such as lung cancer (12, 22). This is important as lung cancer is the most common type of cancer globally, and cigarette smoking is responsible for up to 90% of lung cancer cases worldwide (23, 24).

Nevertheless, the research question for this review was “can exercise combined with NRT promote smoking cessation and reduce withdrawal symptoms in adult smokers?” A preliminary search of MEDLINE, the Cochrane Database of Systematic Reviews, Scopus, EMBASE and the JBI Evidence Synthesis was carried out, and there were no current systematic reviews on the topic identified. Thus, this systematic review is important as it is the first to examine the effects of aerobic- and resistance-based exercise in conjunction with NRT for smoking cessation. This differs from previous systematic reviews that specifically explore the impact of exercise as an intervention for smoking cessation (25, 26), but a combined effect of exercise and NRT. In this review, exercise interventions ranged from home-based to laboratory-based, incorporating exercises of different intensities. While most studies focussed on moderate-high intensity aerobic-based exercises, some studies chose a more “lifestyle” approach, encouraging activities with moderate intensity, such as walking. While regular exercise can exert long-term benefits in successful quitting attempts, most evidence is derived from short-term studies (12, 27, 28). Studies on long-term exercise and smoking cessation are limited, likely due to the difficulty of maintaining longer-term exercises, especially high-density and frequent ones. However, the urge to smoke is also typically experienced by smokers following abstinence and deters successful quit attempts (16). While NRT results in some declines in cigarette consumption (29), exercise-aided NRT cessation programmes are likely to be more successful in achieving abstinence and further drop the plateaued smoking rates.

## Methods

### Search strategy

In this review, a three-step search strategy was implemented to find published studies (Table 1). Initially, a preliminary search in PubMed and Medline using pre-selected search terms was conducted to analyse titles, abstracts and index terms. A second search was performed using identified search terms and keywords across all included databases. Then, a final examination of the reference lists within included studies was conducted to identify any papers that may have been missed. Only papers in English were included. A date limitation of papers published since 2000 was imposed on search strategies.

**TABLE 1:** Search performed in different databases on 22nd September 2022.

Database	Search number	Query	Filters	Results
PubMed	1	((((((((((physical activity) OR (exercise)) OR (fitness)) AND (cessation)) OR (stop)) OR (quit)) AND (nicotine replacement)) OR (replacement therapy)) OR (NRT)) OR (nicotine replacement therapy)) OR (replacement)	From 2001 to 2022	411,515
	2	((((physical activity) OR (exercise)) AND (cessation)) OR (smoking)) AND (nicotine replacement)	From 2001 to 2022	3,729
	3	((exercise) AND (smoking cessation)) AND (nicotine replacement therapy)	From 2001 to 2022	70
Embase	1	(((((physical activity or exercise or fitness) and cessation) or stop or quit) and nicotine replacement) or NRT or nicotine replacement therapy or replacement).af.	From 2001 to 2022	372,097
	2	((((physical activity or exercise) and cessation) or smoking) and nicotine replacement).af.	From 2001 to 2022	6,985
	3	(exercise and smoking cessation and nicotine replacement therapy).af.	From 2001 to 2022	199
Cochrane	1	(physical activity) OR (exercise) OR (fitness) AND (cessation) OR (stop) OR (quit) AND (nicotine replacement) OR (NRT) OR (nicotine replacement therapy) OR (replacement) with Cochrane Library publication date Between January 2001 and September 2022	From 2001 to 2022	198,015
	2	(physical activity) OR (exercise) AND (cessation) OR (smoking) AND (nicotine replacement) with Cochrane Library publication date Between January 2001 and September 2022	From 2001 to 2022	67,987
	3	(exercise) AND (smoking cessation) AND (nicotine replacement therapy) with Cochrane Library publication date Between January 2001 and September 2022	From 2001 to 2022	70
Medline	1	((((physical activity or exercise or fitness) and cessation) or stop or quit) and nicotine replacement) or NRT or nicotine replacement therapy or replacement).af. limit 1 to year = “2000–2022”	From 2000 to 2022	278,980
	2	((((physical activity or exercise) and cessation) or smoking) and nicotine replacement).af. limit 3 to year = “2000–2022”	From 2000 to 2022	8,293
	3	(exercise and smoking cessation and nicotine replacement therapy).af. limit 5 to year = “2000–2022”	From 2000 to 2022	53

Databases used in this review included PubMed, Medline, Embase and Cochrane Central Register of Controlled Trials (CENTRAL). Search terms consisted of the following: “nicotine replacement therapy,” “exercise,” and “smoking cessation.” The keywords included “Cigarette smoking,” “exercise,” “cessation,” “nicotine replacement therapy.”

### Inclusion criteria

#### Participants

Male and female tobacco smokers who wished to quit, or recent quitters, between the ages of 18 and 70 years. Mental disorders were not excluded, as exercise can improve mental health status. Therefore, it may be as effective among people with mental health issues as the general population in preventing nicotine cravings and supporting abstinence.

#### Intervention(s)

Studies involving exercise and NRT, alone or in conjunction with other smoking cessation interventions, were included.

#### Comparator(s)

Studies comparing exercise-aided NRT interventions to standard smoking cessation interventions or other types of non-exercise interventions were included.

#### Outcomes

Studies with the following outcomes were included: smoking cessation, reduced cigarette cravings, and reduced nicotine intake. In the case of multiple cessation measures, continuous and prolonged cessation were preferred over point-prevalence cessation, although all types were considered. Biochemically validated measures of abstinence were also preferred over self-reported measures of abstinence, although both methods were included.

#### Types of studies

This review considered all qualitative studies on the effects of physical activity with/or NRT on smoking cessation.

### Study screening and selection

All selected studies were imported into EndNote (Clarivate Analytics, PA, USA) with duplicates removed. Two independent reviewers (YY and HM) screened the titles and abstracts to ensure that the references met the inclusion criteria. Studies with abstracts and titles that met the inclusion criteria were included. Those with insufficient information had the full texts screened to determine whether they can be included. Any discrepancies in study screening and selection were resolved with a third reviewer (HC or CY). Citations were then exported to the JBI System for the Unified Management, Assessment and Review of Information (JBI SUMARI; JBI, Adelaide, SA, Australia).^1^

### Critical appraisal

Prior to inclusion, two independent reviewers assessed (HC and YY) eligible studies for methodological quality. Reviewers followed the JBI Critical Appraisal Checklist for Randomised Controlled trials (Supplementary Figure 1). The JBI checklist for randomised controlled trials is considered reliable and valid, comprising thirteen items. For each item, studies were assigned one of four ratings: yes (high methodological quality), no (low methodological quality), unclear, or not applicable. Any discrepancies were resolved by discussion between the reviewers, and then by consultation of a third reviewer (CY or BGO) if a resolution was not met.

### Data extraction

Data were extracted and summarised using the JBI Data Extraction Form for Experimental/Observational Studies (Supplementary Figure 2). Extracted data includes participant demographics, inclusion and exclusion criteria, study design and follow-up of interventions, and outcome measurements. Discrepancies between reviewers were resolved through discussion and consultation with a third reviewer.

### Data synthesis

This review adopted a broad search strategy and included clinically diverse studies. A narrative synthesis of the results was performed. A description of the included studies, results and methodological quality is presented in Table 2.

**TABLE 2:** Study characteristics.

References, Country	Participants mean age (SD), sex	Exercise conditions	Setting	Sample size	Duration
Al-Chalabi et al. (36), United Kingdom	*Intervention* 32.5 (12.6) years, 50% female *Control* 36.5 (10.9), 55% female	Isometric exercises and body scan	Smoking cessation clinic	40	4 weeks
Arbour-Nicitopoulos et al. (33), Canada	50.14 (9.45) years, 57.1% female	10-min passive sitting, and 10-min brisk walking	Clinic	14	<1 week
Bernard et al. (39), France	*Intervention* 8.5 (10.9) years, 48.6% female *Control* 48.4 (10.0) years, 68.6% female	The supervised exercise sessions: a 5-min warm-up, followed by 30 min of aerobic activity (stationary cycle ergometer) and a 5-min cool-down period including stretching. The home exercise sessions: 40 min of aerobic exercise (i.e., walking, cycling, or running).	Clinic	70	8 weeks
Bize et al. (40), Switzerland	42.2 (10.1) years, 57% male	Moderate-intensity physical activity: (a) interactive discussion on physical activity (5 min); (b) a warm-up period, a 45-min physical activity session; (c) debriefing (5–10 min).	University, recreational centre	481	9 weeks
Chaney and Sheriff (30), United States	Age not reported, 100% female	A structured exercise programme: an 8-week programme of physical activity with an exercise plan that increased physical activity each week by 30 min.	community recreational centres	101	8 weeks
Dunsiger et al. (32), United States	42.5 years, 100% female	Moderate intensity exercise: three 50-min exercise sessions (3 min of warm-up and 3 min of cool-down for a total of 56 min per session) per week for 12 weeks.	Laboratory, Community	105	12 weeks
Harper et al. (19), Canada	41.29 (13.28) years, 100% female	Moderate intensity exercise: aerobic exercise performed on a variety of equipment that included treadmills, rowing ma-chines, stair climbers, or stationary bicycles	Home, Laboratory	178	14 weeks
Harper et al. (34), Canada	42.01 (13.02) years, 100% female	Moderate intensity exercise: a 14-week exercise programme at the Exercise and Health Psychology Laboratory (www.ehpl.uwo.ca) at Western University.	Laboratory	149	14 weeks
Jonsdottir and Jonsdottir (41), Iceland	Control 4.7 (11.2) years, 38.2% male 42.8 (12.6) years, 61.8% female Intervention 9.5 (9.8) years, 63.6% male 38.7 (7.8) years, 36.4% female	Supervised exercise programme 3 times a week for 2 months and self-organised for 4 months. started with 40 min and gradually increased to 80 min 3 times a week. aerobic training (40%, treadmills and stationary biking), weight lifting (40%), and stretching exercises (20%).	HealthCare Centre and health club	67	1 year
Maddison et al. (38), New Zealand	Intervention 37.6 (12.2) years, 54.3% female Control 37.5 (12.2) years, 54.1% female	The Fit2Quit intervention	Home, Community	906	24 weeks
Prapavessis et al. (37), New Zealand	*Intervention* 37.9 (12.4) years, 100% female *Control* 38.2 (10.9) years, 100% female	Supervised exercise on a cycle ergometer, treadmill, and rower, with the majority of each session spent on the cycle ergometer.	Laboratory	142	12 weeks
Prapavessis et al. (12), Canada	*Exercise* + *smoking cessation maintenance* 41.96 (12.7) years, 100% female *Exercise maintenance* + *contact control* 43.47 (14.02) years, 100% female *Smoking cessation maintenance* + *contact control* 43.45 (12.22) years, 100% female *Contact control* 40.36 (11.92) years, 100% female	Exercised in a supervised facility at the Exercise and Health Psychology Laboratory (EHPL) and used various cardiovascular machines, such as treadmills, rowing machines, stair climbers and stationary bicycles. Participants’ workload (intensity and duration) progressively increased to 70–75% of their maximum heart rate over the 14-week period as the number of exercise sessions tapered.	Home, Laboratory	413	56 weeks
Smits et al. (31), United States	*Standard smoking cessation treatment* 45.39 (11.30) years, 54.7% female *Exercise* + *Standard smoking cessation treatment* 43.12 (11.26) years, 50% female	Participants completed a 5-min warm-up and 25 min of exercise, followed by a 5-min cool-down. Facilitators monitored heart rate during each session and adjusted treadmill speed and/or incline to ensure participants trained at the target heart rate	Laboratory	136	15 weeks
Tritter et al. (28), Canada	*Intervention* 38.64 (8.25) years, 20% male *Control* 41.73 (12.1), 46% female	Based on the short-form Intentional Physical Activity Questionnaire, participants’ physical activity were classified as low, moderate or high.	Laboratory	30	<1 week
Ussher et al. (17), United Kingdom	*Exercise* 41.5 (11.1) years, no sex ratio *Control* 44.4 (11.1) years, no sex ratio	30 min of moderate-intensity exercise on 5 days a week, or 20 min of vigorous exercise on 3 days a week during the past 3 months.	Community	299	7 weeks
Ussher et al. (35), United Kingdom	The same cohort as the above study in 2003.				
Williams et al. (27), United States	*Intervention* 41.5 (12.3) years, 100% female *Control* 43.3 (11.0) years, 100% female	Three sessions per week of brisk walking for 50 min session, thus equalling the recommended 150 min of moderate intensity aerobic exercise per week. All exercise was performed on treadmills.	Laboratory	60	8 weeks

## Results

### Study inclusion

**FIGURE 1:**
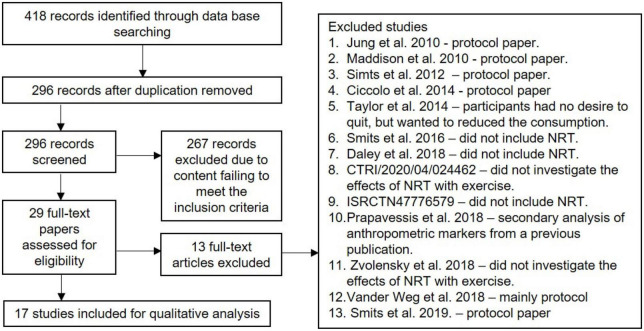
PRISMA flow diagram.

This review considered all types of quantitative research studies on the effects of physical activity with NRT on smoking cessation. Figure 1 summarises the inclusion and exclusion process. There were 17 studies selected for this review (Table 2). Four studies were performed in the USA (27, 30–32), four in Canada (12, 19, 28, 33, 34), four in the UK (12, 17, 35, 36), two in New Zealand (37, 38), one in France (39), one in Switzerland (40), and one in Iceland (41).

### Quality of methodology

The risk of bias assessments using the JBI Critical Appraisal Checklist for randomised controlled trials (Supplementary Figure 1). Overall, the quality of the methodology of the included studies were moderate to high. However, the methodology in four studies was low-moderate quality (12, 27, 30, 42) and one used self-referred grouping rather than randomised by the researchers (41).

All studies except for three claimed to use randomisation method. Most studies reported similar baseline characteristics between groups, while three did not indicate baseline comparisons (19, 30, 34). Nine studies performed allocation concealment, while this was unclear in five studies (12, 27, 28, 30, 35). None of the studies blinded participants as this was not possible for exercise interventions, but two studies blinded the therapists (32, 36). Four studies reported blinding the assessors performing outcome measurements (19, 34, 36, 42) and the ten studies did not specify (12, 27, 28, 30–33, 35, 37, 39). The remaining 3 studies did not blind the outcome assessors. Among the included studies, experimental groups were treated identically in only five studies (28, 30, 36, 38, 41). These studies compared the effects of smoking cessation support with or without exercise. Follow-up was performed for 12 studies, while it was unclear for four studies (27–29, 31) and incomplete for one study (30). Eleven studies used an intent-to-treat approach, while the remaining did not (12, 27, 28, 30, 33, 36). All studies measured the same outcomes for all experimental groups. Most of the studies used reliable outcome measures; however, the reliability of outcome measures in three studies was unclear (30, 33, 38). Additionally, whilst the authors of this study have closely looked at the statistical approach taken in the initial studies and did not find anything of concern, we would encourage readers of this review also to look at their methodology before embarking on studies of a similar nature. All studies used appropriate trial designs.

### Characteristics of included studies

#### Participants

The included studies had a total of 3,191 participants. The largest study was a two-arm randomised controlled trial with 906 participants (38). However, seven studies had less than 50 participants per treatment group (27–29, 33, 36, 37, 41). Three studies were considered pilot trials (30, 33, 36). Fourteen studies randomised current smokers interested in quitting (12, 17, 19, 27, 30–32, 34–40), and one randomised recent quitters (28). Three studies had participants with mental illness (31, 33, 39), while the remaining studies investigated smokers from the general population. Six studies specifically recruited female participants (12, 19, 27, 30, 32, 37). One study reported predominantly Caucasian participants (40), and one predominantly Maori participants (38).

Six studies did not include participants with low physical activity levels (19, 28, 30, 33, 34, 36). The average age of participants in four studies was below 40 years (28, 36–38), and that in ten studies was above 40 years (12, 19, 27, 30–34, 39, 40). One study included participants below and over 40 years of age (41), and one study did not specify the age in the inclusion criteria (30).

#### Intervention(s)

All eligible studies varied considerably in treatment durations, three studies between 0 and 5 weeks (28, 33, 36), six studies between 5 and 10 weeks (17, 27, 30, 35, 39, 40), six studies between 10 and 15 weeks (12, 19, 31, 32, 34, 37), and two study greater than 20 weeks (38, 41).

All seventeen studies included NRT in their smoking cessation programmes. Among these studies, 13 used NRT for both intervention and control groups (12, 27, 28, 30–33, 36, 38–41, 43), while two did not have a control group and used NRT in conjunction with an exercise programme (19, 34). In these two studies, the participants served as their own controls to compare the parameters before and after the exercise in a pre/post study design. Two studies had 4 comparison arms (12, 37).

Eight studies used NRT in the form of a nicotine patch (12, 17, 31, 32, 34, 35, 37, 41) and four studies used NRT in the form of a lozenge (19, 27, 28, 30). Five studies used a combination of NRT products (33, 36, 38–40), and two studies used pharmacotherapies, such as varenicline and bupropion (33, 39).

Different doses of NRT products were used in different studies according to local guidelines. Most studies adopted a gradual dose titration system (12, 28, 37). One study provided 15 mg nicotine patches (35), while two studies provided 21 mg patches throughout the programme (19, 34). Two studies tailored the NRT dose to the baseline cigarette consumption of the participants (32, 41). One study used 2 and 4 mg lozenges (30), and another study used 2 and 4 mg nicotine gum, 10 mg nicotine inhaler and 1 mg lozenge (40). In the study by Bernard and colleagues, participants received a dose of 0.5 mg varenicline daily, which was gradually increased over the course of study (39). Six studies did not specify the dosage (27, 31, 33, 36, 38, 39). As NRT doses may need to be titrated in different patients according to their daily smoking consumption, those studies were not excluded.

Regarding exercise type, most of the included studies used aerobic-based exercises. However, some studies used home-based exercise programmes. One study used both resistance and aerobic-based training (30). One study performed supervised exercises (aerobic and stretching exercises) for 2 months, followed by self-administered exercise for another 4 months (41). One study focussed on telephone counselling to promote exercise and smoking cessation maintenance following the completion of the programme (12); and one focussed on telephone counselling sessions to promote exercise behaviour (38).

Six studies began the intervention prior to the quitting day (12, 31, 34, 37, 39, 40); two began on the quitting day (32, 41); and four began after the quitting day (19, 28, 36, 38). Five studies did not specify when the intervention began relative to the quit date (17, 27, 30, 33, 35).

One study found that smoking status consistently correlates to adherence, with abstainers having higher exercise adherence rates than smokers (12). Similarly, another study found that the number of exercise intervention calls significantly reduced the rate of smoking (38). One study found nearly doubled abstinence rate with additional exercise (39.4%) compared with NRT alone (20.6%) at 1 year, albeit with no statistical significance (*P* = 0.16) (41). Among the included studies, three involved participants’ goal setting (17, 31, 38). Six studies incorporated self-monitoring (17, 30, 31, 33, 37, 40). Two studies used reinforcement strategies of some kind (12, 38). Bernard and colleagues encouraged non-attenders over the phone to continue and schedule a make-up session (39). Three studies used exercise maintenance programmes (12, 30, 38). Two studies used cash incentives to encourage participants to attend sessions (31, 32).

Attendance rates varied considerably among the studies. Bernard et al. demonstrated high attendance (82%) at group sessions and moderate attendance (67%) at home-based sessions (39). In another study, participants had an average attendance of 55% throughout the 14 weeks of supervised exercise (12). In the study by Al-Chalabi and colleagues, 75% of participants performed the weekly isometric exercises (36). Three studies reported lower attendance rates in the exercise intervention group than in the control intervention (31, 32, 37). The other studies did not report attendance rates.

Of the eligible studies, eight relied on self-reported changes in physical activity levels (12, 17, 30, 33, 37–40). Among the studies comparing physical activity levels between the exercise and control groups, six reported significantly higher physical fitness for the exercise group than the control group (31, 32, 35, 37, 39, 40). Another study observed an increase in self-reported physical activity levels in both exercise and control groups, with participants in the exercise group spending more time being active in their leisure time (38). Only one study measured fitness objectively using an accelerometer (39), and the same study also reported better fitness in the exercise group (39).

Six studies reported some but no significant differences in abstinence rates between intervention and control groups (12, 32, 35, 37, 39, 40). Prapavessis et al. reported consistently higher smoking cessation rates with the addition of NRT to both exercise and cognitive behavioural therapy groups (37). Two studies reported non-significantly higher continuous abstinence rates among control groups compared with the intervention groups (36, 38), whereas one study reported a non-significantly higher abstinence rate in the exercise group (39.4%) than the control (20.6%) at 6 months follow up (41). In the study by Ussher et al. the exercise group had non-significantly higher rates of continuous abstinence at 6 weeks, but not at 12 months (35). Two studies found significantly higher cessation rates in the intervention group than in the control group (30, 31). Regarding cigarette cravings, two studies found reduced cravings in both the experimental and control groups, with a greater magnitude for the experimental group (28, 33). Similarly, two studies reported significant reductions in cravings from pre- to post-exercise (19, 34). Only one study found no sustained effects of exercise on cravings (27). One study reported significantly less weight gain in the control group vs. the intervention group (30); while another study reported significantly greater weight gain in participants who abstinent from smoking compared with those still smoking in the exercise group, not the control group (41). The weight gain could be attributed to lean mass rather than fat. However, another study reported lower average weight gain among participants in the exercise group (40).

#### Comparator(s)

Eleven studies included smoking cessation support comparators (17, 27, 28, 30–32, 36–40). One study included relapse prevention support comparators (12). One study had passive sitting as the comparator (33). Of all the studies, fifteen studies provided cessation support for both intervention and control groups, and two studies provided this support for control groups only (33, 37).

The mode of delivery of these support sessions varied among studies, with one study delivering *via* telephone (12), six *via* face-to-face contact (27, 30, 32, 36, 39, 41), and one *via* both telephone and face-to-face contact (38). Four studies did not specify the mode of delivery (31, 35, 37, 40).

Fourteen studies set a quit day for current smokers (12, 19, 28, 31–41). Two studies did not appear to set a quit date (27, 30).

Nine studies compared exercise in conjunction with smoking cessation support with cessation support alone (27, 30, 32, 35–39, 41), and reported the effects of exercise as an add-on strategy on smoking cessation. On the other hand, the study using relapse prevention support did not provide clear evidence of the effects of exercise on smoking cessation (12).

#### Outcome(s)

Among the selected studies, three measured continuous abstinence (12, 35, 39) and two measured prolonged abstinence (36, 41). One study measured both point prevalence and prolonged abstinence (31) and four studies measured both point prevalence and continuous abstinence (32, 37, 38, 40). Six studies did not specify the measurement of abstinence (19, 27, 28, 30, 33, 34).

Abstinence was validated by expired-air carbon monoxide levels in nine studies (12, 19, 27, 28, 34–36, 39, 40), and by saliva cotinine in one study (38). Three studies used both expired-air carbon monoxide and saliva cotinine levels (31, 32, 37). Three studies did not specify the method of validation (30, 33, 41).

## Discussion

Findings from this review suggest that exercise, in conjunction with NRT, is an effective intervention for smoking cessation in the short term. However, the efficacy was generally reduced in the medium- and long-term. It is possible that exercise can only be maintained under supervision for the duration of the study to support the short-term abstinent effects.

This review included 17 trials to determine the effectiveness of an NRT-aided exercise intervention in promoting smoking cessation. Interventions in these studies included both aerobic- and resistance-based exercises. Of all the included studies, nine reported some positive effects of combined exercise and NRT in promoting smoking cessation (12, 19, 27, 28, 31, 34, 36, 37, 41). A previous Cochran review also showed no strong evidence of long-term efficacy of exercise on relapse in two prevention studies (25). Similarly, a meta-analysis found that aerobic exercise was successful in smoking cessation intervention during the first 3 months of cessation, with no difference from behavioural treatment in the medium- and long-term follow-ups (26). Smoking cessation is strongly related to behavioural and lifestyle changes, which can explain why participants experienced similar longer-term effects from behavioural treatment and exercise. NRT, on the other hand, has demonstrated clear efficacy on abstinence in both short-term (44) and the long-term (45) due to the control of nicotine craving. However, the efficacy of NRT in the long-term can be achieved by a single course of treatment, although the relapse rate can reach 30% at 12 months (45). Exercise with NRT may increase the short-term abstinent rate; however, continuous exercise beyond the treatment period may be needed to sustain such effect, which is difficult to achieve without supervision.

In terms of the health benefits of exercise, a single bout of exercise can improve executive functions, mood, and stress levels (46). This is because such exercise can induce changes in the brain regions that can improve long-term memory and motor functions (46). As such, the general recommendation is 150 min of moderate-intensity or 75 min of vigorous-intensity exercise every week to sustain such effects (47). However, high-intensity exercises induce more positive health effects than low-intensity exercises, such as improved cardiorespiratory fitness and reduced oxidative stress and inflammation (48). Most studies included in this review examined the effects of a moderate-high intensity exercise, which not only promoted smoking cessation rate, but also improved withdrawal symptoms, weight gain, levels of stress, anxiety and depression, therefore increasing the chance of abstinence.

High-intensity exercise can alleviate depression and anxiety in smokers to promote the success rate of quitting (49); however, it is also difficult to be maintained. Indeed, adherence heavily influences the efficacy of exercise. Among the included studies, two reported that high adherence positively affected smoking cessation (31, 38). Therefore, future study design should also focus on increasing exercise adherence not just during the intervention period but also beyond to enhance the success rate of smoking cessation.

Among the eligible studies, six specifically recruited female participants (12, 19, 27, 30, 32, 37), while the remaining recruited mixed genders. In recent years, despite the global downward trend in cigarette use, consumption among women has been growing (50). Some studies have shown that women have more difficulty maintaining long-term abstinence than men, although the reasons for this are unclear (51). However, this only applies to those aged 50 and above, with the opposite trend in those below 50 (52). Two studies provided the rationale for choosing females only in their trials (19, 32). Firstly, the efficacy of NRT in women is not as good as in men. Therefore, it is more difficult for women to quit and maintain abstinence after quitting smoking. Secondly, women seem more concerned about weight gain after quitting than men; therefore, a quitting method that can effectively prevent craving and weight gain would encourage more quitting attempts among female smokers. Social support among women with the same intention of quitting smoking may also help with the outcome. Among the mixed-gender studies in this review, there were no gender differences in the effect of exercise and NRT on abstinence.

### Strengths and limitations

This is a comprehensive review of trials that evaluated the efficacy of combined exercise and NRT in promoting smoking cessation. Our methods are reproducible. Two reviewers screened the studies and extracted the data. JBI Critical Appraisal Checklist for Randomised Controlled Trials was used to assess the risk of bias, and Joanna Briggs Institute’s (JBI’s) GRADE approach was followed to assess the certainty of evidence. Additionally, the included studies were randomised controlled trials (except for three study), which are less susceptible to bias than other study types. This review also has several limitations. Firstly, most studies were in high-income countries, such as the USA and Canada; whereas medium-lower income countries generally have a higher prevalence of smoking. Secondly, participants were primarily from the general population. More research is needed in special populations whose ability to quit and adhere to exercise differs from the general population, e.g., people with disabilities, especially those with mental health issues. The third limitation of this review is the broad inclusion criteria of the participant’s age. Exercise adherence is largely dependent on age, and this should be considered in future reviews. The fourth limitation is the self-reporting nature of the method used in most studies. As a result, such data can be inaccurate. There might be recall bias, and some unreported lifestyle choices may also affect the efficacy of quitting interventions and thereafter study outcomes. Finally, the search strategy was limited to English publications. Therefore, it is possible that trials published in other languages were omitted.

## Conclusion

Physical activity has the potential to increase the success of smoking cessation in the short term, when used in conjunction with behavioural support and NRT. While incorporating exercise with NRT has a minimum effect on abstinence in long-term settings, it is important to note that adherence to long-term exercise is difficult without close supervision. Future studies should develop strategies to increase exercise adherence to promote long-term abstinence.

## Data availability statement

The original contributions presented in this study are included in the article/Supplementary material, further inquiries can be directed to the corresponding author.

## Author contributions

HC and BGO: conceptualisation and methodology. HC, YY, HM, CY, and BGO: investigation. CY and BGO: supervision. HC and YY: writing—original draft. All authors: writing—review and editing.

## Footnotes

1
https://sumari.jbi.global/
