# Supplementary material for: The effects of exercise with nicotine replacement therapy for smoking cessation in adults: A systematic review
Source: Front Psychiatry. 2022 Nov 24;13:1053937. doi: 10.3389/fpsyt.2022.1053937 (PMC9730281; doi:10.3389/fpsyt.2022.1053937)

**The effects of exercise with nicotine replacement therapy for smoking cessation in adults: a systematic review**

Hui Chen ^1^, Yang Yang^2^, Hanna Miyai ^1^, Chenju Yi ^2^, Brian G Oliver ^1,3^

1. School of Life Sciences, Faculty of Science, University of Technology Sydney, Ultimo, NSW 2007, Australia
2. Research Centre, The Seventh Affiliated Hospital of Sun Yat-sen University, Shenzhen, 518107, China.
3. Respiratory Cellular and Molecular Biology, Woolcock Institute of Medical Research, The University of Sydney, Glebe, NSW 2037, Australia

**Correspondence**

Associate Professor Chenju Yi, Research Center, The Seventh Affiliated Hospital, Sun Yat-sen University, 628 Zhenyuan Road, Guangming, Shenzhen, Guangdong 518107, China. Email: [yichj@mail.sysu.edu.cn](mailto:yichj@mail.sysu.edu.cn)

**Supplementary Figure 1: Critical appraisal instrument**


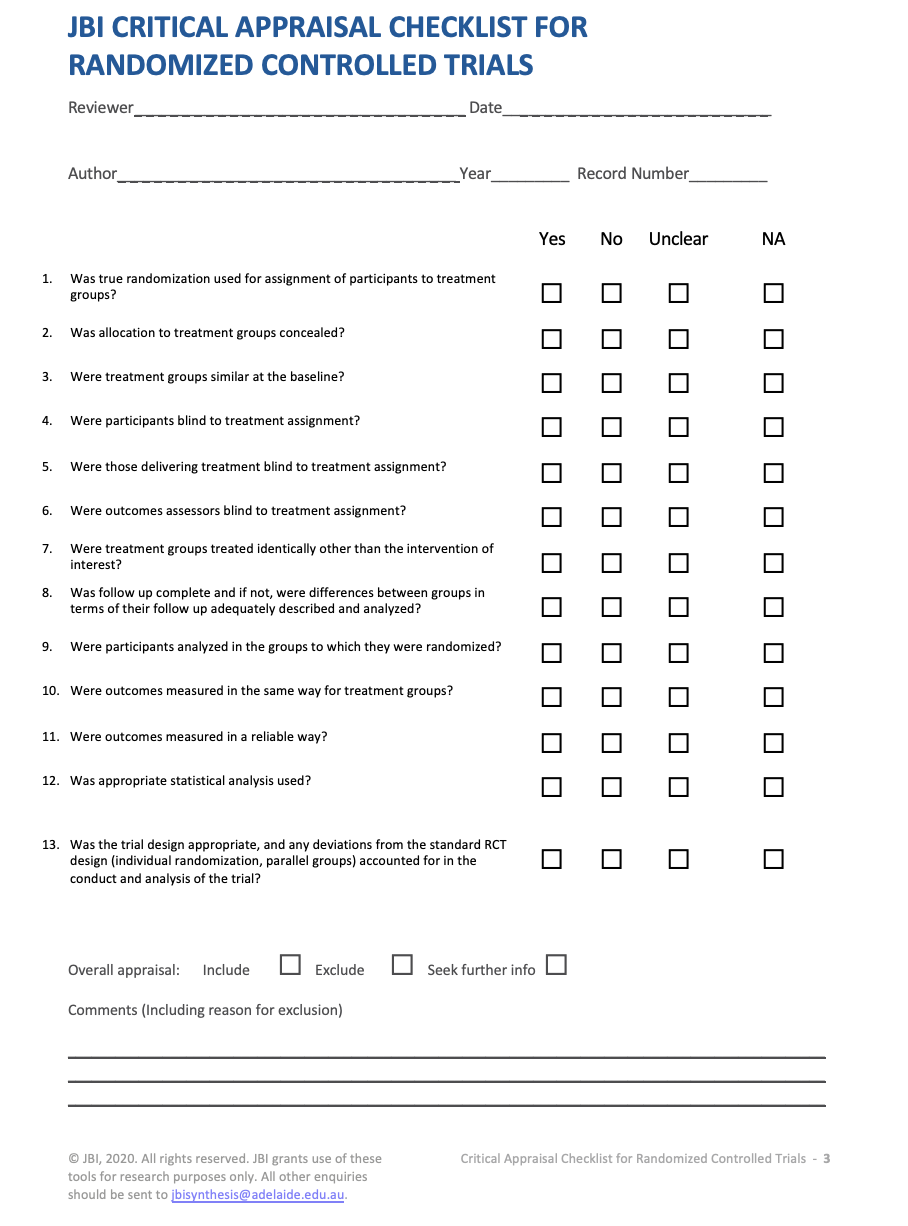


**Supplementary Figure 2: Data extraction tool**


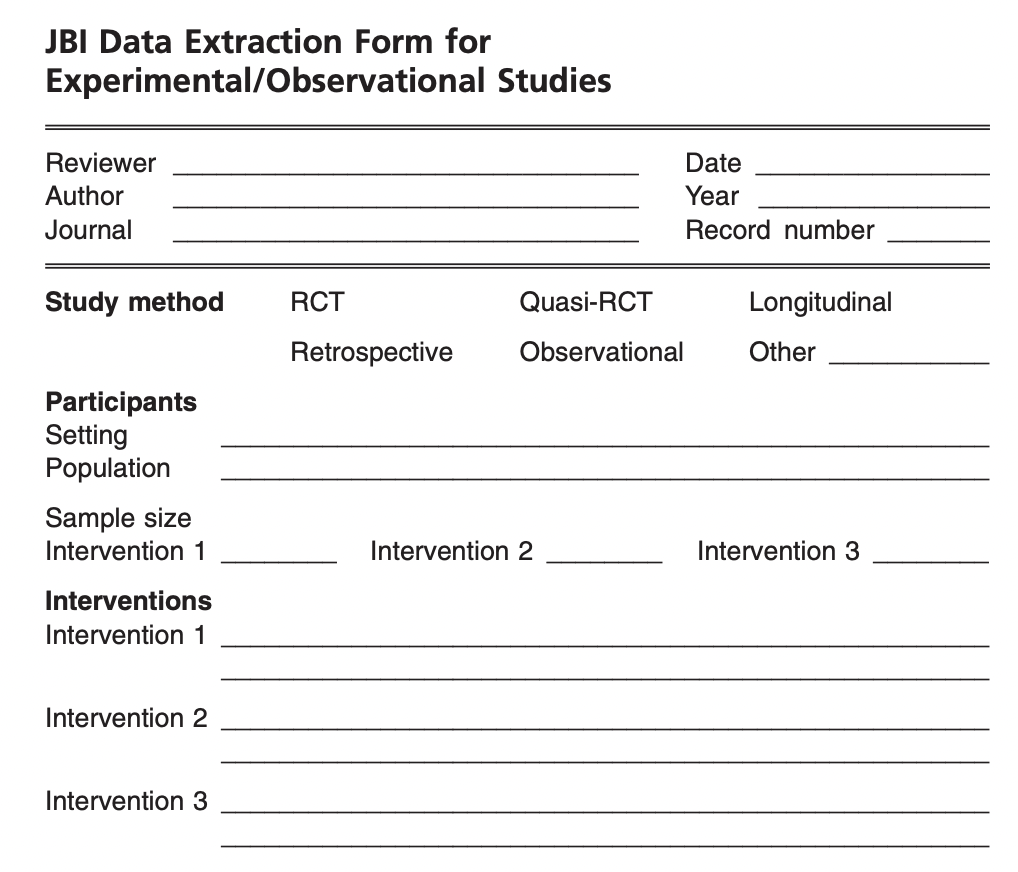


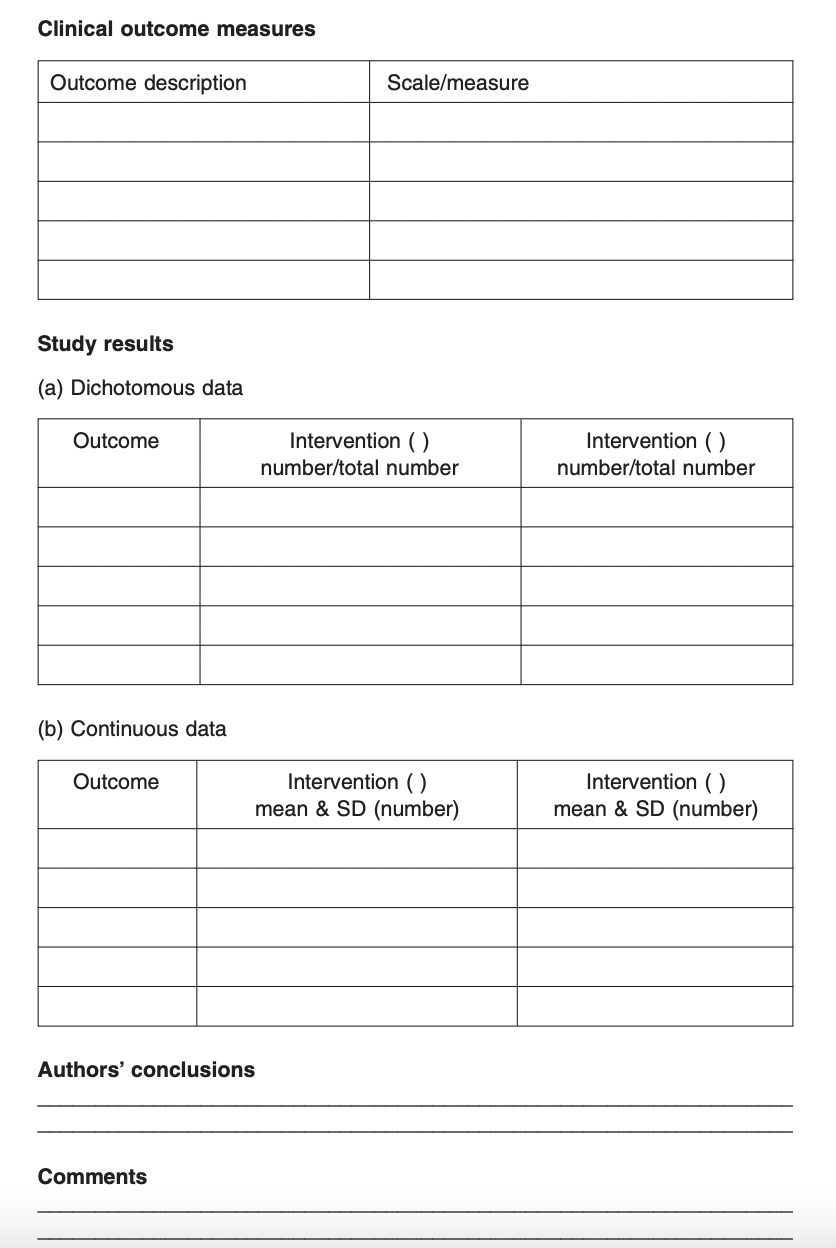

Supplement: Supplementary file 1 [file Data_Sheet_1.docx]
